# A cryptic Allee effect: spatial contexts mask an existing fitness–density relationship

**DOI:** 10.1098/rsos.150034

**Published:** 2015-06-03

**Authors:** Akira Terui, Yusuke Miyazaki, Akira Yoshioka, Shin-ichiro S. Matsuzaki

**Affiliations:** 1 Department of Forest Science, Graduate School of Agriculture, Hokkaido University, Kita 9, Nishi 9, Kita-ku, Sapporo 060-8589, Japan; 2 Kanagawa Prefectural Museum of Natural History, 499 Iryuda, Odawara, Kanagawa 250-0031, Japan; 3 National Institute for Environmental Studies, Tsukuba-shi, Ibaraki 305-8506, Japan

**Keywords:** Allee effect, multilevel model, freshwater mussel, lotic system, stream

## Abstract

Current theories predict that Allee effects should be widespread in nature, but there is little consistency in empirical findings. We hypothesized that this gap can arise from ignoring spatial contexts (i.e. spatial scale and heterogeneity) that potentially mask an existing fitness–density relationship: a ‘cryptic’ Allee effect. To test this hypothesis, we analysed how spatial contexts interacted with conspecific density to influence the fertilization rate of the freshwater mussel *Margaritifera laevis*. This sessile organism has a simple fertilization process whereby females filter sperm from the water column; this system enabled us to readily assess the interaction between conspecific density and spatial heterogeneity (e.g. flow conditions) at multiple spatial levels. Our findings were twofold. First, positive density-dependence in fertilization was undetectable at a population scale (approx. less than 50.5 m^2^), probably reflecting the exponential decay of sperm density with distance from the sperm source. Second, the Allee effect was confirmed at a local level (0.25 m^2^), but only when certain flow conditions were met (slow current velocity and shallow water depth). These results suggest that spatial contexts can mask existing Allee effects.

## 1. Introduction

Declines in individual fitness with decreasing conspecific density, termed Allee effects, can be an important determinant for the risk of extinction of small populations [1]. A variety of mechanisms give rise to Allee effects (e.g. decreased reproductive efficiency at low conspecific density) and current theories predict that they may be pervasive in nature [2]. However, there is little consistency in empirical findings [3–5] and considerable debate exists as to why evidence from natural populations is under-represented in some taxa [6]. This gap is problematic because failure to detect true Allee effects may cause an overly optimistic assessment of any threats [2].

Historically, most studies on the Allee effect took place in homogeneous spaces (e.g. [7]) and assumed a ‘constant’ fitness–density relationship in any aggregation (but see [8,9]). Although this simplification makes complex density-dependent processes tractable, it compromises at least two general properties of nature. First, any spatial scale under consideration may be important in detecting true Allee effects. The outcomes of ecological processes are often scale-dependent, such that data gathered at an inappropriate spatial scale may fail to show patterns that exist at a smaller or larger level [10]. Second, spatial heterogeneity in environmental conditions can interact with density-dependent processes. For example, many sessile organisms rely on their surrounding media (water or air) for gamete transfer [11], so flow or wind conditions may modify the fitness–density relationship by interfering with fertilization processes [12]. Under these circumstances, spatial contexts could mask an existing fitness–density relationship: a ‘cryptic’ Allee effect. This cryptic nature could explain the scarcity of evidence from natural populations.

Freshwater mussels (Bivalvia: Unionoida) are undergoing a catastrophic decline worldwide, partly due to their complex life cycle [13]; the larvae (glochidia) are obligate parasites on the gills or fins of fish [14]. Despite this, mussels serve as an excellent organism to examine the potential interactions between spatial contexts and density-dependence because they are sessile and make discrete aggregations known as mussel beds (populations) [14,15]. These factors allow us to readily define the scale of interactions and patchiness in conspecific density [10]. Moreover, their fertilization process is sensitive to variations in conspecific density and flow conditions (i.e. spatial heterogeneity): their eggs are deposited in the gills of the female mussel where they are fertilized by sperm filtered from the water column [16]. Female mussels at low conspecific density may receive fewer gametes, as sperm aggregates may diffuse rapidly after being released from male mussels [17]. Such a phenomenon is particularly relevant to lotic environments in which the dilution of gametes may be extreme.

Here, we examined how spatial contexts interact with conspecific density to influence the fertilization success of the Japanese riverine mussel *Margaritifera laevis* (classified as ‘vulnerable’) [18], which inhabits high-gradient streams with fast currents and coarse substrata. The potential interaction between conspecific density and environments that affect flow conditions (e.g. current velocity; hereafter ‘flow-mediating environments’) was evaluated at population (approx. less than 50.5 m^2^) and local levels (0.25 m^2^). Two predictions were made based on previous empirical knowledge: (i) the effects of conspecific density on fertilization success would be apparent at the local level, as sperm density is likely to decrease exponentially with distance from the sperm source [17,19]; (ii) faster current velocity, deeper water depth and coarser substrate would make the existing Allee effect obscure because those conditions may facilitate the dispersion of sperm and homogenize the sperm availability within the population [12,20,21]. We acknowledge that positive density-dependence in fertilization (i.e. component Allee effects) may not necessarily have substantial impacts on population growth rate (i.e. demographic Allee effects; *sensu* [22]), but its quantification is an essential first step to explore the possibility of demographic Allee effects.

## 2. Material and methods

### 2.1 Study area and species

The investigations were conducted in the Shubuto River system, located near Kuromatsunai, Hokkaido Prefecture, Japan (42°40′ N, 140°18′ E). The mean annual temperature and precipitation were 7.4°C and 1461.8 mm, respectively. The water catchment area encompasses approximately 367 km^2^, and the length of the main river is approximately 40 km. The host fish *Oncorhynchus masou masou* was abundant and widely distributed throughout the river system [23,24]. The water quality was suitable for most freshwater organisms throughout the river system (dissolved oxygen more than 95% saturation, pH 7.0–8.1, biochemical oxygen demand 0.5–1.7 mg l^−1^ and ammonia concentration 0.05–0.13 mg l^−1^) [15,25].

In the Shubuto River system, the brooding period of *M. laevis* begins in mid-June and lasts for approximately one month [26]. Developed larvae (glochidia) are released from early to mid-July and infect the gills of the host fish (mainly parrs) with an extremely high prevalence near the dense mussel beds (approx. 100%). This parasitic stage lasts for 40–50 days [27]. Juveniles with shell lengths ranging from 0.3 to 0.6 mm detach from the host fish during late summer and disperse passively via the river currents. Sexual maturity occurs at approximately 8–13 years of age [28], and their maximum lifespan is approximately 79 years [29]. *Margaritifera laevis* is the only species of freshwater mussel within the riverine network and has no known predators (e.g. crayfish and muskrats).

**Figure 1.**
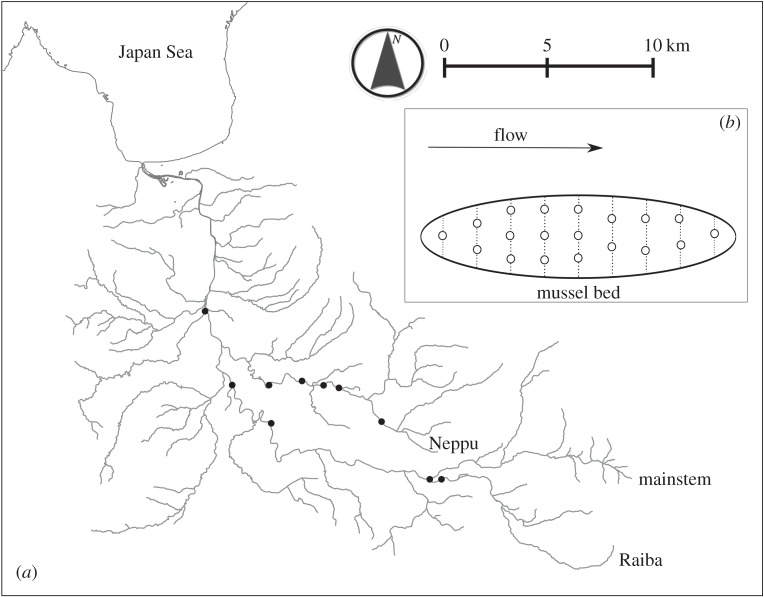
(*a*) Map of the Shubuto River system. Filled dots indicate sampling sites. (*b*) Schematic diagram of mussel sampling design. Dotted lines and open plots indicate sampled transects and locations of gravid females sampled, respectively.

We studied 10 populations from three rivers: the Shubuto (*n*=3), Neppu (*n*=5) and Raiba rivers (*n*=2), where a 1:1 sex ratio was validated in preliminary surveys (A.T. 2012, unpublished data; figure 1*a*). The populations were separated from each other by at least 600 m. These populations have different mussel bed sizes (1–50.5 m^2^) and were located more than 500 m apart from the nearest large population [15].

### 2.2 Egg collection and fertilization analyses

The investigation was conducted from 27 June to 20 July in 2012 and on 27–28 June in 2013, when fertilized and unfertilized eggs could be differentiated more easily (A.T. 2012, personal observation). Gravid females were sampled from 10 populations in 2012 and four populations in 2013 (i.e. 14 population replicates with 10 unique populations). For each population, a qualitative visual search was carried out with a glass-bottomed viewing bucket to locate the approximate boundaries of the population. The boundaries were defined based on gaps between local aggregations of adult mussels that exceed 20 m. Demographics of those aggregations were assumed to be independent to some extent as mussel larvae rarely dispersed more than 20 m via host fish [26]. An initial transect, oriented perpendicular to the water flow, was placed at the lowest end of the population, and additional transects were placed at 3–5 m intervals upstream (figure 1*b*). Transects were added until the upstream extent of the population had been sampled.

In each transect, adult mussels were haphazardly selected (more than 40 mm in shell length), and their shells were gently pried open to check for the presence of inflated gills (an indication of brooding) [28]. Approximately one to three gravid females were collected from each transect (figure 1*b*) and were placed separately into a polyethylene bag. The collection locations of the gravid females were marked by metal pegs in the substrate.

The shell lengths of the collected gravid females were measured with callipers, and the eggs were extracted using a syringe for fertilization analysis. About 40% of the gill contents (i.e. eggs) were extracted from the dorsal, middle and posterior parts of the left and right gills. The extracted eggs were flushed into a numbered collection bottle (50 ml) and fixed with 70% ethanol for transportation back to the laboratory. The gravid females were released back into the populations from which they came after collection of the egg samples.

In the laboratory, the egg samples were flushed onto a zooplankton counting plate after gentle shaking to distribute the contents evenly. The numbers of developing glochidia, and fertilized and unfertilized eggs were counted, until a combined total of 100 units was reached. This procedure was repeated three times for each gravid female, and the fertilization rate was calculated as the number of fertilized eggs and glochidia present in the total number of eggs and glochidia counted (300 units). The presence of a fertilization membrane surrounding an egg distinguished fertilized eggs from unfertilized eggs (O. Kobayashi 2012, personal communication). Glochidia were easily identified by the presence of shell valves. A preliminary analysis showed no significant difference in the fertilization rate between the syringed and whole egg samples, both of which were obtained from the same 15 fixed gravid females (paired Wilcoxon test, *p*>0.8), validating the use of the syringed samples for further analyses.

### 2.3 Environmental factors and adult mussel density

Concurrent with the collection of gravid females, environmental factors that could potentially mediate the flow conditions at a local level (current velocity, water depth, substrate coarseness) were measured at the locations where the gravid females were collected. The current velocity was measured with a flow meter (VE20, VET-200-10PII; Kennek, Tokyo, Japan) and water depth was measured using a measuring rod at the locations of the sampled gravid females (i.e. where the metal pegs had been placed). The current velocity was measured for 30 s for three repeats, and the average measurement was used for statistical analysis. Substrate coarseness was measured according to a modified method of Inoue & Nakano [30]. A quadrat was placed (0.25 m^2^) at the locations of the collected gravid females, and the proportion of each of the following categories was visually estimated: particles less than 2 mm=sand, 2–16 mm=gravel, 17–256 mm=cobble and 256–1024 mm=boulder. These categories were then coded as follows: sand=1, gravel=2, cobble=3 and boulder=4. The substrate coarseness of the quadrat was calculated by the following equation: substrate coarseness=Σ(material code×the proportion of the category).

As a final step, local conspecific density was quantified in each quadrat. After collecting all the visible mussels, the substrate within a quadrat was excavated to a depth of 10 cm using a hand-trowel and shifted through a 2 mm mesh sieve to separate mussels from the sediment. Mussels with a shell length more than 40 mm were considered to be sexually mature [28], and the number of adult mussels was recorded.

At a population level, catchment area was used as a flow-mediating environment because it affects population-level flow dynamics [31] and was calculated using ArcGIS 10.0 (Esri, Redlands, CA, USA) with Digital Map 25 000 (National and Regional Planning Bureau), at a scale of 1:25 000. Mean population density, a measure of conspecific density at the population level, was estimated as the mean of the local conspecific density for each population.

### 2.4 Statistical analyses

To simultaneously assess the interacting effects between conspecific density and flow-mediating environments on fertilization success at two spatial scales (population: approx. less than 50.5 m^2^, local: 0.25 m^2^), a hierarchical linear model was implemented within a Bayesian framework [32,33]. In the model, the number of fertilized eggs of gravid female *i* (*y*_*i*,*j*,*t*_) recorded in population *j* and year *t* was assumed to follow a binomial distribution, *y*_*i*,*j*,*t*_∼Binomial (*n*_*i*,*j*,*t*_,*p*_*i*,*j*,*t*_). The parameter *p*_*i*,*j*,*t*_ is the expected fertilization rate of gravid female *i* in population *j* and year *t*. The total number of eggs counted (*n*_*i*,*j*,*t*_) is constant for all the gravid females (*n*=300). The expected fertilization rate is related to linear predictors via a logit-link function as

$$\begin{array}{rl} \mathrm{logit}(p_{i,j,t}) & =\alpha_{j,t}+\beta_{1}\cdot {\mathrm{DEN}}_{i,j,t}+\beta_{2}\cdot {\mathrm{VEL}}_{i,j,t}+\beta_{3}\cdot {\mathrm{DEP}}_{i,j,t}+\beta_{4}\cdot {\mathrm{SUB}}_{i,j,t}+\beta_{5}\cdot {\mathrm{DEN}}_{i,j,t}\cdot {\mathrm{VEL}}_{i,j,t}\\ & \;+\beta_{6}\cdot {\mathrm{DEN}}_{i,j,t}\cdot {\mathrm{DEP}}_{i,j,t}+\beta_{7}\cdot {\mathrm{DEN}}_{i,j,t}\cdot {\mathrm{SUB}}_{i,j,t}+\beta_{8}\cdot {\mathrm{LOC}}_{i,j,t}+\beta_{9}\cdot {\mathrm{SHELL}}_{i,j,t}+\gamma_{k(j),t}+\varepsilon_{i,j,t}, \end{array}$$

where *α*_*j*,*t*_ is a population-specific intercept term and *β*_1−9_ are regression coefficients for local conspecific (adult) density DEN_*i*,*j*,*t*_, local current velocity VEL_*i*,*j*,*t*_, local water depth DEP_*i*,*j*,*t*_, local substrate coarseness SUB_*i*,*j*,*t*_, location of a gravid female LOC_*i*,*j*,*t*_, shell length of a gravid female SHELL_*i*,*j*,*t*_ and their interactions. Location of a gravid female (distance (m) from the upstream edge of the population) was incorporated in the model to reduce the potential for spurious associations driven by increased sperm density in the downstream portion of the populations. Shell length of a gravid female was also included to account for body-size variations among individuals. The random effect *γ*_*k*(*j*),*t*_ for the sampling date *k*(*j*) nested within year *t* enabled the model to account for random variations in the fertilization rate during the sampling dates and years (*k*(*j*) indicated the sampling date of the investigation in population *j*). The random effect *γ*_*k*(*j*),*t*_ was assumed to follow a normal distribution as $\gamma_{k(j),t}∼\mathrm{Normal}\;(0,\sigma_{\gamma }^{2})$. The other term *ε*_*i*,*j*,*t*_ was a random effect to account for overdispersion and was assumed to follow a normal distribution as $\varepsilon_{i,j,t}∼\mathrm{Normal}\;(0,\sigma_{\varepsilon }^{2})$ [34].

The population-specific intercept was modelled as a random effect with a mean determined by a global intercept *α*_global_, and population-level covariates as follows:

$$\alpha_{j,t}=\alpha_{\mathrm{global}}+\alpha_{1}\cdot P\_{\mathrm{DEN}}_{j,t}+\alpha_{2}\cdot {\mathrm{CAT}}_{j,t}+\alpha_{3}\cdot P\_{\mathrm{DEN}}_{j,t}\cdot {\mathrm{CAT}}_{j,t}+\omega_{j,t},$$

where *α*_1−3_ are regression coefficients for mean population density *P*_DEN_*j*,*t*_, catchment area CAT_*j*,*t*_, and an interaction between *P*_DEN_*j*,*t*_ and CAT_*j*,*t*_. The error term was normally distributed as $\omega_{i}∼\mathrm{Normal}\;(0,\sigma_{\omega }^{2})$. It is important to note that the effects of the population-level variables were inferred using all the data points (91 females; see Results) [32]. Thus, this model provides robust estimates of the population-level effects despite our modest sample size at the population level (14 population replicates).

Vague priors were assigned for the parameters: i.e. normal distributions with a mean of 0 and variance of 100 for *β*_1−9_, *α*_global_, and *α*_1−3_, and uniform distributions ranging from 0 to 100 for $\sigma_{\gamma }^{2}$, $\sigma_{\varepsilon }^{2}$ and $\sigma_{\omega }^{2}$. Correlation coefficients for all combinations of explanatory variables were less than 0.5.

The goodness of fit of the hierarchical model was evaluated by the Bayesian *p*-value [34]. A Bayesian *p*-value close to 0.5 was taken as an indication of a good model performance, whereas a value close to 0 or 1 was taken as an indication of poor model performance.

The model was fitted to the data with OpenBUGS v. 3.2.2 and R2OpenBUGS in R v. 2.15.3 [35]. The number of Markov chain Monte Carlo steps, burn-in and thin were 100 000, 20 000 and 20, respectively. Convergence was assessed by examining whether the R-hat indicator of each parameter had reached a value close to 1 [32].

## 3. Results

### 3.1 Fertilization rate and surrounding environments

Ninety-nine gravid females (49 individuals, 2012; 50 individuals, 2013) were collected and their fertilization rates were investigated; however, discerning between fertilized and unfertilized egg samples in eight individuals was difficult and thus discounted from further analyses. Consequently, the numbers of females collected from each population ranged from 1–12 and 4–20 individuals in 2012 and 2013, respectively, from a total of 91 individuals. Their shell lengths ranged from 53 to 114 mm, with a mean length of 78.0 mm. In both sampling years, the fertilization rate varied greatly among the gravid females from less than 0.05 to 1.0. The values of hypothesized influential variables varied widely across the study sites at the population and local levels (table 1).

**Table 1.** Values of hypothesized influential variables at 10 mussel populations.

scale	factor	mean (s.d.)	range
population	mean population density^a^ (ind./0.25 m^2^)	18.4 (11.0)	0.5–31.8
	catchment area (km^2^)	77.6 (63.5)	19.1–252.6
local	local conspecific density (ind./0.25 m^2^)	18.9 (18.6)	0–79
	current velocity (cm s^−1^)	16.5 (14.4)	0.2–76.5
	water depth (cm)	29.7 (13.6)	10–62
	substrate coarseness^b^	2.7 (0.3)	1.3–3.3

^a^Mean population density was estimated as the average of the local conspecific density for each study population.

^b^Substrate coarseness was assessed following the modified method of Inoue & Nakano [30]. See text for details.

### 3.2 Factors influencing the fertilization rate of *Margaritifera laevis*

**Figure 2.**
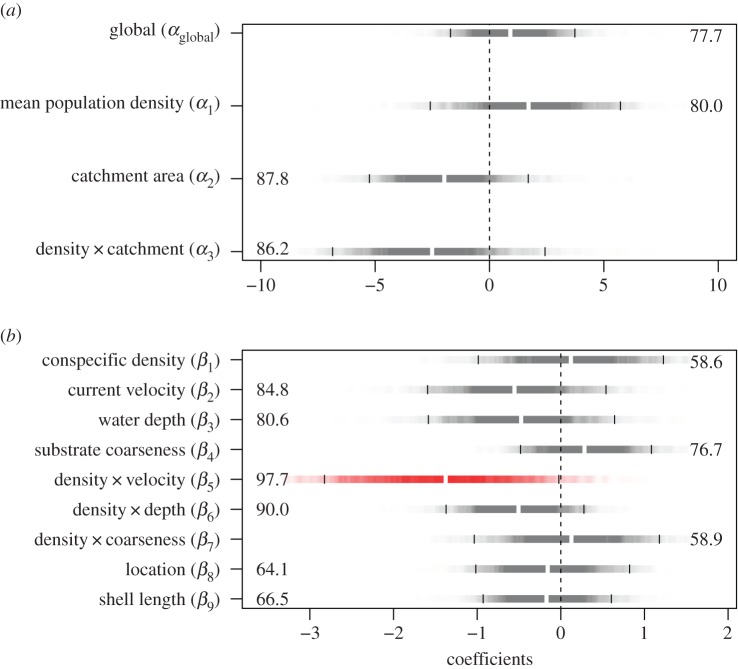
Estimated parameters for the hierarchical model explaining the fertilization rate of *M. laevis* at (*a*) the population and (*b*) the local level. Horizontal bars are shaded in proportion to the posterior probability density, and white and black vertical lines mark the median estimates and 95% credible intervals, respectively. Parameters for which 95% credible intervals did not include zero are shown in red. Numbers in the panels indicate the probability of being either positive or negative for each parameter. All explanatory variables were standardized before the analysis.

**Figure 3.**
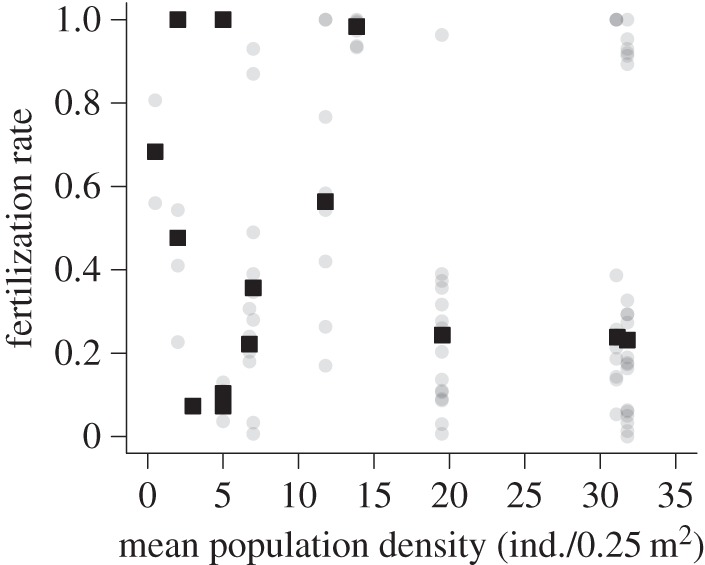
Little influence of population-level conspecific density on the fertilization rate of *M. laevis*. Black squares and grey circles indicate observed values of fertilization rate for each population (median) and each gravid female, respectively. Mean population density was estimated by averaging local conspecific density for each study population.

The hierarchical model revealed a clear contrast in the effects of the hypothesized influential variables between the spatial levels. At the population level, none of the variables (mean population density, catchment area and their interaction) had significant effects on the fertilization rate of *M. laevis* (figure 2*a*). The fertilization rate was scattered, irrespective of the mean population density (figure 3).

**Figure 4.**
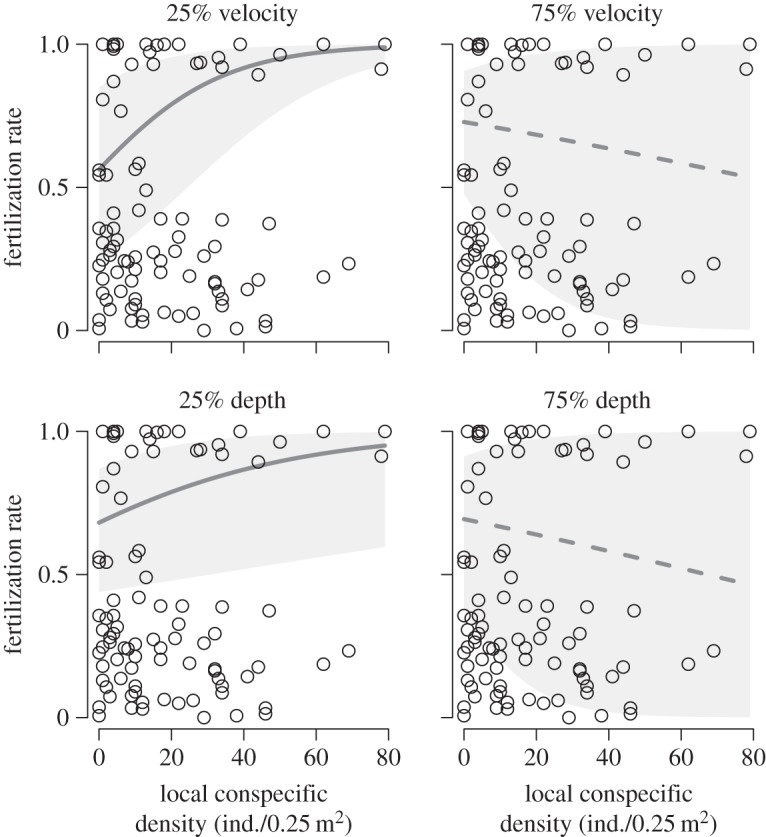
Local conspecific density interacts with current velocity and water depth to influence the fertilization rate of *M. laevis*. Solid (significant) and broken lines (not significant) indicate median prediction derived from the hierarchical linear model fitted to the data, and shaded area shows 95 percentile of prediction. Predicted values are shown separately at 25 and 75 percentiles of current velocity and water depth. Other explanatory variables not shown on the panels were fixed at mean values for prediction. Circles indicate observed values.

By contrast, local conspecific density strongly interacted with the flow-mediating variables to influence the fertilization rate (figures 2*b* and 4). The fertilization rate rose clearly with increasing conspecific density in places with slow currents, but the relationship diminished as the current velocity increased (figure 4). Although the interacting effect of local water depth was not significant in terms of the 95% credible interval, its probability of being negative exceeded 90% (figure 2). The positive effect of local conspecific density disappeared as the water depth increased (figure 4). Substrate coarseness and its interaction with local conspecific density had little influence on the fertilization rate (figure 2). Location and shell length of the gravid females were unlikely to influence the fertilization rate as their credible intervals had a wide range, starting at 0 (figure 2).

The estimated Bayesian *p*-value for the hierarchical model was 0.48. This indicates that the model performance was reasonably good. However, the estimated dispersion parameter ($\sigma_{\varepsilon }^{2}$) was 8.4, suggesting that unknown variables that were accounted by the random effect (i.e. *ε*) played a role.

## 4. Discussion

The Allee effect did act on the fertilization process of *M. laevis*, but it was easily masked by spatial contexts. The use of an inappropriate spatial scale, in this case the population level, made it difficult to detect positive density-dependence. Furthermore, even at an appropriate spatial scale (i.e. local level), the existing Allee effect became less apparent when certain flow conditions were not met (slow current velocity and shallow water depth).

While many theoretical and empirical studies have suggested that Allee effects in reproduction may be widespread in nature [4,11], contradictory results were also observed from natural populations. Myers *et al*. [5] found evidence for Allee effects in only three of 128 fishery stocks, and a recent meta-analysis reported similar patterns across a variety of taxa [6]. Freshwater mussels are no exception; some studies have examined the relationship between fertilization success and conspecific density in natural populations [17,36,37], but only one of them confirmed the existence of Allee effects [17]. To date, this lack of evidence has been attributed to ‘very few data at low abundance’ [38]. However, our findings suggest another possible explanation for why Allee effects have been under-represented in natural populations: Allee effects can be hidden by complex interactions with spatial contexts.

The physics of the dilution process may explain the difference in the effects of conspecific density between the spatial scales. Previous studies have reported that the fertilization rate of marine sessile organisms decreased exponentially with distance from the sperm source [19], suggesting that the spatial extent of sperm transfer through the medium (ocean currents) is limited. This probably holds true for our study system because the sperm transfer of *M. laevis* depended completely on the water currents [27]. Observations on increased aggregations of freshwater mussels during the spawning season [39] also suggest limited sperm dispersal, further supporting our interpretation. Hence, population-scale variables not fully explaining the variation in the fertilization rate of *M. laevis* is understandable.

At the local level, the existing Allee effects were detectable only when the current velocity was sufficiently slow, which is not unexpected as swift currents may facilitate the dispersion of sperm [20] and homogenize the sperm availability within the habitat. Another possible mechanism is that current velocity influenced the filtering efficacy of female mussels. However, if this were the case, the main effect of current velocity should be positive, as fast current velocity promotes the resource capture of suspension feeders from the water column [21,40–42]. Thus, increased dispersion of sperm aggregates is probably the primary mechanism behind the negative interaction between conspecific density and current velocity.

Likewise, marginal interacting effects with local water depth may be due to the increased homogenization of sperm density in deeper areas. Babcock *et al*. [12] showed a slight decline in the fertilization rate of the sea star *Coscinasterias muricata* with increasing water depth, and their mechanical models suggested that water depth facilitates the dilution of sperm clouds. That a similar phenomenon occurred in *M. laevis* is reasonable because the experimental settings and mechanical models mimicked the gamete dilution process of broadcast spawners in general [12].

The limited influence of the substrate coarseness on the fertilization rate was unexpected, given that local topographic variations can induce complex water flows and thereby influence resource capture of suspension feeders [21,43]. One possible explanation is that the filtering position of *M. laevis* is sufficient for it to avoid near-bed turbulence arising from bed-form complexity. Adult *M. laevis* are usually located approximately 1–5 cm above the substrate (A.T. 2012, personal observation), where the local topographic variation is less likely to influence the flow conditions [43].

Our results should be viewed with some caution. First, the population replicates were limited in number, which could cause low statistical power at the population scale. Although our statistical approach is robust against this problem (i.e. using information from all the individuals to infer the effects of population-level variables), we acknowledge that this possibility is unavoidable. Second, we focused on a single fitness component (fertilization), so whether our findings could be scaled up to a population level (i.e. population growth rate) is unclear. Currently, the cryptic Allee effect may act on a limited proportion of a population and can have minor effects on the population dynamics. Nevertheless, ongoing habitat modifications (e.g. artificial flow regulation) may cause the effects to become much more pervasive across a population and the Allee effect can shift from being ‘cryptic’ to being ‘apparent’. Although further exploration will be required to assess the consequences, *a priori* awareness of cryptic Allee effects would help avoid changes in environmental conditions that might lead to escalating positive density-dependence.

Our study was limited in its taxonomic extent, like most empirical studies, but the interplay between Allee effects and spatial contexts may be widespread in nature. Many aquatic and terrestrial sessile organisms (e.g. echinoderms and plants) rely on water currents or wind for the transfer of their gametes [19,44], whereby spatial components are likely to interact with density-dependent processes in reproduction. Accumulating evidence indicates that mating behaviour and animal-mediated pollination are influenced by biotic and abiotic environments [8,45,46]. These facts imply that our hypothesis may be broadly applicable to many organisms. Researchers should acknowledge the uncertainty associated with spatial contexts in uncovering cryptic Allee effects; ignoring this complexity could provide erroneous information for management strategies.

## Data accessibility

Data were archived on Dryad: http://dx.doi.org/10.5061/dryad.bb75p.
